# BRAIN-Diabetes: a randomised trial to test the feasibility of an adapted FINGER multidomain intervention in adults with type 2 diabetes living in rural border regions of Ireland

**DOI:** 10.1007/s10433-025-00862-0

**Published:** 2025-06-24

**Authors:** Claire T. McEvoy, Geraldine McCarthy, Rebecca F. Townsend, Catherine Dolan, Joanne Regan-Moriarty, Christopher Cardwell, Bernadette McGuinness, Seán P. Kennelly, Jim Kelly, Catherine McHugh, Frank Kee, John Bartlett, Caroline Bradshaw, Orla Reynolds, Valerie Mortland, Christina O’Neill, Ingrid McLoughlin, Noel McCaffrey, Margaret Heffernan, Cabrini Nolan, Peter A. Passmore

**Affiliations:** 1 The Centre for Public Health, Institute of Clinical Sciences B, School of Medicine, Dentistry and Biomedical Sciences, Queen’s University Belfast, Belfast, Northern Ireland UK; 2 The Global Brain Health Institute, University of California San Francisco, San Francisco, USA; 3 National University of Ireland, Galway and Sligo Leitrim Mental Health Services, Sligo, Ireland; 4 School of Biomedical, Nutritional and Sport Sciences, Faculty of Medical Sciences, Newcastle University, Newcastle Upon Tyne, UK; 5 Department of Health and Nutritional Sciences, Atlantic Technological University, Sligo, Ireland; 6 Institute of Memory and Cognition, Tallaght University Hospital, Dublin, Ireland; 7 Department of Medical Gerontology, School of Medicine, Trinity College Dublin, Dublin, Ireland; 8 South West Acute Hospital, Western Health and Social Care Trust, Enniskillen, Northern Ireland UK; 9 Department of Endocrinology and Diabetes, Sligo University Hospital, Sligo, Ireland; 10 ExWell Medical, Irish Wheelchair Association, Dublin, Ireland; 11 Physiotherapy Department, Sligo University Hospital, Sligo, Ireland

**Keywords:** Multidomain intervention, Feasibility, Cognitive performance

## Abstract

**Background:**

The Border Region Area lifestyle INtervention for healthy cognitive ageing in Diabetes’ (BRAIN-Diabetes) trial aimed to test the feasibility of an adapted version of the Finnish Geriatric Intervention Study to Prevent Cognitive Impairment and Disability (FINGER) multidomain intervention in cognitively healthy adults at risk of dementia living in border regions of Ireland.

**Methods:**

BRAIN-Diabetes was a 6-month randomised controlled pilot trial involving adults living in rural border regions who were ≥ 50 years old, without existing dementia but had a diagnosis of type 2 diabetes and access to a computer. Individuals were randomised to either the multidomain intervention or the standard care control group. The intervention included diet counselling, physical exercise and computerised cognitive training which were delivered remotely and cardiometabolic risk monitoring which was delivered in person. The primary outcomes assessed feasibility of recruitment/retention and adherence to the intervention. Other outcomes explored intervention effects on cognitive, metabolic and health-related quality of life.

**Results:**

In total, 156 individuals were assessed for eligibility, and 79 (51%) were recruited (mean age 61.6 ± 6.9 (range 60–75) years; 68% male). After 6 months, retention was 81% (72% in intervention versus 90% control). Adherence rate was high with most participants attending > 50% of the scheduled intervention sessions. There was greater improvement in diet quality (*p* < 0.001), daily step count (*p* = 0.04), triglyceride levels (*p* = 0.02) and health-related quality of life (*p* < 0.05) in the intervention group compared to control. There were no observed intervention effects on cognitive performance over 6 months.

**Conclusions:**

The BRAIN-Diabetes pilot trial demonstrated that an adapted FINGER model was feasible to deliver and efficacious in supporting lifestyle behavioural changes among a unique at-risk rural population. There were also indicative benefits for metabolic health and health-related quality of life over a short time frame.

*Trial registration* ClinicalTrials.gov (registration ref: NCT05304975 accepted 31st March 2022).

**Supplementary Information:**

The online version contains supplementary material available at 10.1007/s10433-025-00862-0.

## Background

Dementia is the result of progressive brain damage that impairs memory and other cognitive functions and the ability to carry out everyday activities of daily living. It is one of the leading causes for years of healthy life lost due to disability in later life (World Health Organisation 2022) and has a high socioeconomic burden. Approximately 100,000 people are living with dementia across the island of Ireland and this figure is expected to double by 2040, unless effective approaches are identified to prevent or delay future dementia cases (National Dementia Office Ireland 2023; Alzheimer’s Society Dementia Strategy for Northern Ireland 2024). Dementia is disproportionately higher in rural regions (Wiese et al. 2023) which may be due to rapid ageing, poverty risk, greater cumulative exposure to cardiometabolic risks and unequal access to health and social care compared with those in better-connected urban regions. Currently, effective strategies to address inequities in brain health promotion are lacking in Ireland. Emerging immunotherapies are promising treatment options but are not widely available for dementia risk reduction due to strict eligibility criteria and serious side effect profile (Togher et al. 2022). Addressing modifiable cardiometabolic risk factors remains the most promising approach for primary dementia prevention (World Health Organisation 2019).

Type 2 diabetes (T2D) is one of the most prevalent metabolic disorders globally and is an established risk factor for dementia (Livingston et al. 2020). People with T2D have over 50% increased risk of developing dementia compared to those without diabetes (Cao et al. 2024). T2D has been associated with accelerated cognitive decline and cognitive impairment particularly in domains of memory and executive function (Sola et al. 2024) due to chronic glycaemic dysfunction, insulin resistance and neuroinflammation. Brain changes associated with dementia are observed early in T2D before the onset of cognitive impairment (Zhang et al. 2022) representing an opportunity for risk modification. To date, only a few lifestyle trials have been conducted and have shown no treatment effects on cognition outcomes (Launer et al. 2011; Espeland et al. 2017; Luchsinger et al. 2017) likely owing to methodological limitations such as the inclusion of young participants with adequate glycaemic control and minimal cognitive decline during follow-up. Further studies are needed to evaluate cognitive effects of non-pharmacological interventions for T2D, particularly those designed to address multiple dementia risk factors simultaneously in T2D and with longer follow-up periods.

The Finnish Geriatric Intervention Study to Prevent Cognitive Impairment and Disability (FINGER, ClinicalTrials.gov: NCT01041989) (Ngandu et al. 2015) was the first large randomised controlled trial (RCT) to show that an intensive multidomain lifestyle intervention programme (nutrition, exercise, cognitive stimulation and social activity, intensive vascular risk factor control) applied in older people (60–77 years) at higher risk for dementia produced significant cognitive benefits after 2 years. These results are promising but need to be reproduced in other high-risk populations where compliance, acceptability and response to the intervention may vary from FINGER participants. World‐Wide FINGERS (WW-FINGERS) is a global initiative of multidomain trials for dementia risk reduction (Kivipelto et al. 2020) to optimise the FINGER model for various cultures and countries to define feasible and effective preventive strategies for diverse at-risk groups. As part of WW-FINGERS network, the ‘Border Region Area lifestyle INtervention for healthy cognitive ageing in Diabetes’ (BRAIN-Diabetes) trial evaluated the feasibility of an adapted version of the FINGER model among older adults with T2D living in rural border regions of Ireland. Adaptions to the FINGER model were informed by qualitative research with the target population (McEvoy et al. 2023) and addressed barriers to engagement including cultural food norms, and access to exercise and computerised cognitive training programmes.

The main objectives of the 6-month BRAIN-Diabetes pilot RCT were to determine: (i) feasibility of recruitment and retention, (ii) adherence to the intervention components; and (iii) behavioural changes in response to the multidomain intervention. We also explored the effect of the 6-month multidomain intervention on clinical outcomes for metabolic health, cognitive performance and quality of life.

## Methods

### Adjustments to study protocol in response to COVID-19 pandemic

BRAIN-Diabetes was originally planned as a 12-month feasibility RCT conducted in two rural sites across the Island of Ireland: one in Northern Ireland (NI) (covering Tyrone/Fermanagh border area) and one in Southern Ireland (RoI) (covering Sligo/Leitrim/West Cavan border area). The aim was to recruit 140 eligible T2D participants (70 from each of the border regions) and randomly allocate them to either the adapted FINGER multidomain intervention or control groups (35 participants in each group at each site). The multidomain intervention included 4 FINGER domains that were adapted for local context: 1. diet counselling based on a culturally tailored MIND diet suitable for people with T2D (Morris et al. 2015), 2. group-based community exercise based on an Irish ExWell commercial programme previously shown to improve fitness and health outcomes in older people (Skelly 2021), 3. computerised cognitive training (CCT) using BrainHQ (© 2020 Posit Science) a flexible platform used in the U.S. POINTER WW-FINGERS trial (Baker et al. 2024) and 4. nurse-led management of cardiometabolic risk factors (diabetes, hypertension, obesity and dyslipidaemia).

The COVID-19 global pandemic caused recurrent lockdowns nationwide during 2020–2021 forcing delay in commencement of BRAIN-Diabetes. Several adjustments were required to the study protocol to ensure the safety of participants and research staff in response to COVID-19 as summarised in the CONSERVE-CONSORT Extension (Orkin et al. 2021) Additional File 1. The group-based activities were removed from the intervention, and the diet, exercise and CCT components were redesigned to be delivered remotely at home. Additional time was required for protocol adaptation, the introduction of COVID-19 risk assessments, redesign and setting up of remote intervention components, hiring and training research staff and obtaining the necessary ethnics and governance approvals, meaning that the trial start date was delayed by ~ 14 months and the follow-up time was shortened from 12 to 6 months in order to meet the timeline for delivery milestones within the available budget. Data collection for BRAIN-Diabetes started in August 2022 at the RoI site and November 2022 at the NI site and was completed in February 2023 at both study sites.

### Study design

**Fig. 1:**
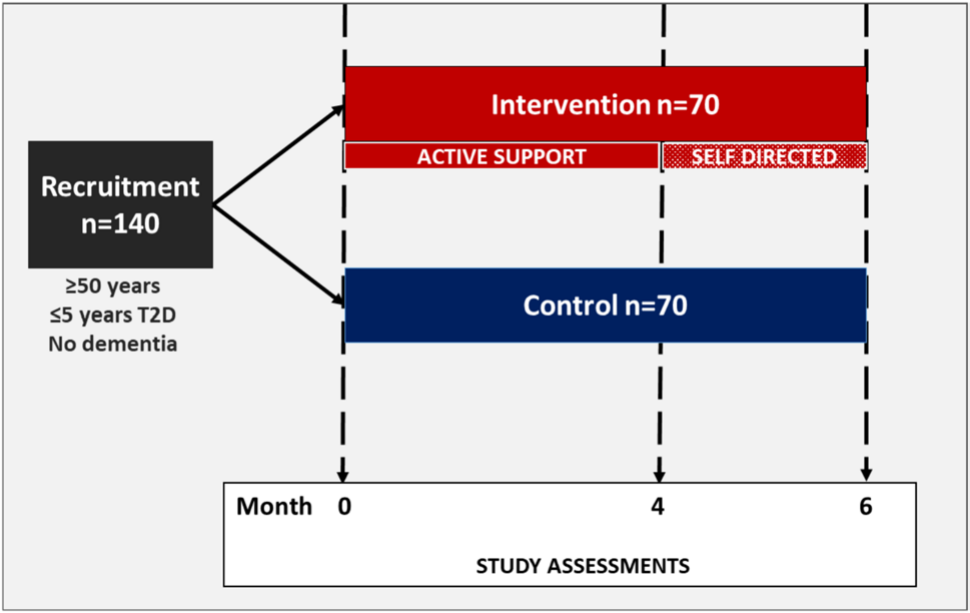
Overview of revised BRAIN-Diabetes design

The revised 6-month parallel group pilot RCT design is shown in Fig. 1. Participants randomised to the control group received standard clinical care while those randomised to the intervention received the multidomain lifestyle programme involving a 4-month active intervention followed by a 2-month self-directed consolidation stage. Study visit assessments were conducted at baseline, 4 and 6 months. Ethical approval for BRAIN-Diabetes was received from the Office for Research Ethics Committees Northern Ireland (REC ref 20/NI/0051) and Sligo University Hospital Research Ethics Committee (775). The study protocol was registered on ClinicalTrials.gov (NCT05304975) and conducted following the Consolidated Standards of Reporting Trials guidelines (CONSORT) extensions for reporting trials modified due to COVID-19 Pandemic (Orkin et al. 2021).

### Screening and recruitment

Several recruitment strategies were employed including diabetes outpatient clinics, media (Facebook advertisement, posters and advertisement in local newspapers) and public outreach events via primary care and diabetes Ireland. Potential participants underwent a screening assessment with a trained nurse at the dedicated research centre or their home according to a strict COVID-19 risk protocol. A personal health and medical history questionnaire and Mini Mental State Examination (MMSE) screener were used to determine eligibility for inclusion in the study. Participants were considered eligible for inclusion if they resided in the rural catchment areas, were ≥ 50 years, had a diagnosis of T2D within the previous 5 years and access to a computer, tablet or smartphone. Individuals were excluded if they were on insulin therapy or had existing dementia or a MMSE score < 20/30 and/or had severe hearing/vision loss, symptomatic cardiovascular disease, untreated depression or other comorbidities that would affect their ability to take part in the study. Informed written consent was obtained from all participants prior to study enrolment.

### Randomisation

Participants were randomly allocated to two groups (Standard Care Control and Multidomain Intervention) in a ratio of 1:1. Randomisation was stratified by study site and used varying block sizes of 2, 4 and 6. A study statistician independent of recruitment, intervention delivery and data collection generated the randomisation sequence in STATA using command RALLOC (Ryan 1997). Allocations were prepared in sealed opaque envelopes by an independent member of the team, and participants were informed of their allocation after the baseline study assessment. It was not possible to blind participants, intervention delivery staff or research nurses to the allocated intervention group. Investigators blinded to treatment allocation conducted data input and cleaning. The study statistician remained blinded to allocation prior to the final analysis.

### Standard care control

Control group participants continued with the standard diabetes care offered by their healthcare provider and received written education at the baseline study visit on brain health along with pedometers to record their walking activity.

### Multidomain intervention

*Diet:* Participants were provided with written dietary education, recipe ideas, tips and suggestions and met online with the study nutritionist for personalised dietary advice. Dietary advice was designed to promote both glycaemic control and brain health for the target Irish T2D population and based on the MIND diet (Mediterranean-DASH diet Intervention for Neurodegeneration Delay) (Additional File 2) recommendations for increasing olive oil, berries, green vegetables, wholegrains, pulses, nuts and fish and reducing intake of fried foods, cheese, butter and confectionery. The MIND dietary food groups were aligned to cultural food norms in Ireland and personalised dietary advice considered individual food preferences and meal patterns. Moderate wine intake was permitted if already being consumed. Participants were supported to set personal dietary goals and to self-monitor progress in changing towards the MIND diet. The intervention target was to increase MIND diet score by at least 2 points. If weight loss was indicated, dietary advice was further tailored to promote 5–10% of body weight reduction. Review appointments took place monthly in the first 4 months either online or by telephone and each lasted up to 60 min. At each review, the nutritionist reinforced the frequency and amount of key MIND diet foods, and discussed progress in changing diet and behavioural strategies to overcome identified challenges in meeting personal dietary goals where applicable.

*Exercise*: Participants received a personalised home-based exercise programme combining aerobic, strengthening and balance exercises based on their baseline physical function. Participants met online with a trained clinical exercise instructor who explained the foundations and safety aspects of the home exercise programme and demonstrated the exercises included within the personal prescription. Most participants started with a basic level of exercise that gradually increased in frequency and intensity over the first four weeks. Participants were encouraged to set exercise goals aiming to perform a minimum of two classes per week at home and to increase walking activity to achieve 10 K daily step count. They were provided with a written resource designed for older adults (ExWell@home), links to video demonstration of the exercises (strength/aerobic/core stability and balance), links to online exercise classes, light dumbbells, a pedometer and an exercise log to track adherence to personal goals. The clinical exercise instructor reviewed participants’ progress on a weekly basis for the first 4 months and provided ongoing support and encouragement to achieve intervention targets.

*Cognitive training*: This component was delivered using Brain HQ® (Posit Science), a CCT platform, which utilises visuospatial and auditory games to enhance attention, mental processing speed, learning and memory. On two occasions during the first 4 weeks participants received an online Brain HQ® cognitive training session delivered by a trained instructor. Additional training support was scheduled on participant request. The Brain HQ® cognitive training application was downloaded on the participants preferred laptop, desktop and/or tablet device. Participants were asked to independently complete the 30-min CCT session at least four days per week at home. Participants received weekly text messages during the first 4 months to provide encouragement and support to complete the CCT sessions.

*Cardiometabolic monitoring*: At each study visit, participants had their blood results (fasting glucose, lipids, glycated haemoglobin (HbA1c)), weight, Body Mass Index (BMI) and blood pressure reviewed. The nurse reinforced the intervention targets and promoted self-management of T2D and vascular risk factors. If required, participants were asked to discuss treatment changes for optimal cardiometabolic health with their primary care provider.

The 4-month active intervention was followed by a 2-month self-directed consolidation phase during which time participants received fortnightly text message reminders regarding the overall multidomain lifestyle intervention targets and monthly phone calls from the study nurse to discuss any issues arising in achieving personal goals and encourage compliance.

### Study assessments

Participants met with a trained research nurse either in their home or a dedicated research facility at baseline, 4 and 6 months for a study visit lasting up to 90 min. A standard protocol was used for study assessments and involved a non-fasting blood sample followed by anthropometric measures of weight (kg) using digital scales (Seca, Germany), height using a portable stadiometer (cm) and waist circumference (cm) using a flexible tape. Clinic-measured pulse rate (beats/minute), and systolic and diastolic blood pressure (mmHg) were measured using a calibrated automated sphygmomanometer (OMRON Healthcare, UK).

A modified neuropsychological test battery (NTB) (Jutten et al. 2020; Lezak et al. 2004) was administered and comprised 10 tests to assess global cognitive performance: the CatchCog (Jutten et al. 2020) Alzheimer’s Disease Assessment Scale–Cognitive subscales (Word Recognition, Immediate Word Recall and Orientation), Controlled Oral Word Association Test, Category Fluency Test, Digit Symbol Substitution Test (DSST) and Digit Span Backward (DSB) supplemented with the Trail Making Tests (A and B) (Lezak et al. 2004) and Delayed learned Word Recall (Lezak et al. 2004). Subdomains of memory were assessed using immediate and delayed word recall test scores, and executive function using Word Association, Category Fluency, Orientation, DSST, DSB and Trails scores.

Physical function was assessed using the Timed Up and Go (TUG) (seconds) to assess functional mobility and balance (Nightingale et al. 2019) and the six-minute walk distance (6MWD) (metres) for aerobic fitness (Nolen-Doerr et al. 2018). A short diet screener (0–15 points) was administered to assess adherence to the MIND diet. Participants also completed a 4-day food diary to assess nutritional intake.

The Short Form (SF-36) 36-item questionnaire (Ware and Sherbourne 1992) was used to assess health-related quality of life (HR-QoL) across physical and mental domains of health status. The physical health score included general health, roles limited due to physical health, physical functioning and bodily pain while the mental health score included mental health, roles limited due to emotional health, social functioning and vitality. Scores range from 0 to 100 with higher scores indicating better HR-QoL.

### Study outcomes

Primary study outcomes were feasibility of recruitment and retention, and adherence with intervention components. Retention was reported as the number and percentage of participants in each group who completed the 6-month follow-up. The reasons for withdrawal were examined for those that dropped out during the trial duration. The original feasibility targets were successful recruitment (at least 75% of pilot study target, i.e. at least *n* = 53) in each site over 18 months at a recruitment rate of 5–6 eligible participants per month; retention of at least 65% in intervention group; and no greater than 20% difference in attrition (proportion of dropouts) between the intervention and control groups. The revised a priori recruitment and retention feasibility criteria were achievement of target recruitment of at least 6 eligible participants per month; retention (proportion of participants completing the 6-month study visit) of at least 75% at 6 months; and 20% or less difference in attrition between the groups. Furthermore, intervention adherence was set as 50% or more participation with scheduled intervention sessions as recorded in study records completed by the research team or logged by the CCT platform. The 50% attendance threshold was selected as the FINGER study showed that 50% attendance in intervention activities was associated with optimal cognitive benefit (Belleville et al. 2022). In addition, diet and exercise behaviour change in the multidomain lifestyle intervention was explored using self-reported change in MIND diet score and logged daily step count.

All secondary outcomes were exploratory and included response in cognitive performance on the NTB global and subdomains, metabolic health measures (BMI, blood lipid profiles and HbA1_C_ level) and HR-QoL. Acceptability measures are reported separately and included interviews with participants to determine acceptability for the intervention components and a study evaluation questionnaire to determine acceptability of data collection procedures and taking part in the trial.

### Statistical analysis

The primary outcomes were feasibility and summarised in descriptive terms and compared to the predefined criteria. Baseline characteristics were determined in the intervention and control groups. Variables were checked for normality before analysis. The MIND diet score and daily step count were compared between the intervention and control group using analysis of covariance (ANCOVA). For example, the difference in mean MIND score at 6 months, adjusting for baseline, was determined using a linear regression model with MIND score at 6 months as the outcome and treatment group and MIND score at baseline as explanatory variables. The same approach was used for daily step count. In each case, an adjusted analysis was conducted also including age, sex and study site in the models. To construct composite cognition scores, the individual raw NTB test scores were standardised to the baseline mean and SD of study participants and then averaged across the number of administered tests. Where applicable, negative z-scores were transformed to positive values to enable the construction of composite scores where higher score reflected better performance. Using this approach, composite z-scores were constructed for global cognition (average across all 10 tests) and domain scores for memory (average across 2 tests) and executive function (average across 7 tests).

ANCOVA, as described above, was used to compare between-group differences in the exploratory secondary outcomes for global cognition, cognitive subdomains, metabolic measures and quality of life scores at 6-month follow-up in completers adjusting for baseline value, study site, age and sex.

## Results

**Fig. 2:**
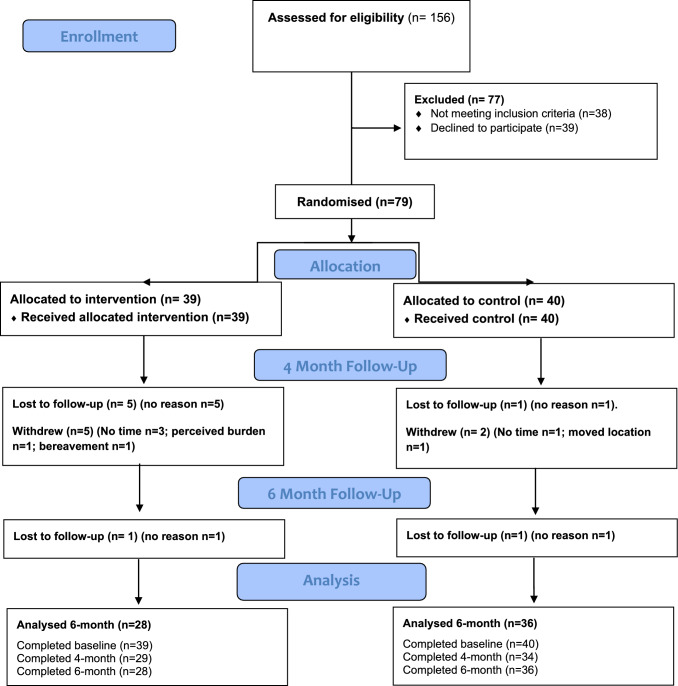
Flow of participants though the BRAIN-Diabetes trial. In the control group *n* = 3, participants did not complete the 4-month follow-up due to illness (*n* = 2) and personal reasons (*n* = 1) but completed the 6-month follow-up

Due to COVID-19, the recruitment period was shortened from 18 to 7 months at the RoI site and 4 months at the NI site. A total of 79 participants were recruited (*n* = 52 in RoI and *n* = 27 in NI) of which 39 participants were allocated to the intervention and 40 participants were allocated to the standard care control group. While the original pre-COVID target recruitment (*n* = 140) was not achieved, recruitment of 7 participants enrolled per month exceeded the revised target. Most participants (89%; *n* = 70) were recruited from outpatient clinic services for T2D, followed by media/advertisement (8%; *n* = 6) and then via public events (3%; *n* = 3). The CONSORT summary of recruited participants, their allocation and reasons for discontinuation/withdrawal from the trial is provided in Fig. 2. After 6 months, overall retention was 81% (72% and 90% in intervention and control groups, respectively) and greater than the minimum 75% feasibility threshold. Overall, there were 15 withdrawals/lost to follow-up with the main reasons being lack of time (*n* = 4), bereavement (*n* = 1) moved location (*n* = 1) and high perceived burden of study (n = 1). Eight participants were unable to be contacted, and no reason was provided. Participants who withdrew or were lost to follow-up were more likely to be younger (mean age 58 ± 5.8) than those who completed the study (62.6 ± 6.8 years) with no other differences observed. There were no adverse events related to study procedures. The difference in attrition rate between the intervention and control groups at 6 months was 18% which was lower than the 20% feasibility threshold.

### Participant characteristics

Baseline participant characteristics according to the allocated group are presented in Table 1. Participants were aged 62.5 ± 6.9 (range 50–75) years, and 69% were male. The mean MMSE score was 28.7 ± 1.26 (range 25–30). All were clinically obese with BMI 32.1 ± 0.68 kg/m^2^ and had reasonable glycaemic control with mean HbA1c 52.1 ± 10.1 mmol/mol. Most participants had normal TUG < 10 s and achieved average 6MWD within the expected range for older adults with T2D (White et al. 2024).

**Table 1:** Baseline characteristics by allocated group

Mean (SD) or n (%)	Standard care control	Multidomain intervention
	(*n* = 36)	(*n* = 28)
Age (years)	63.4 (7.1)	61.4 (6.6)
Female	9 (25)	11 (39)
Weight (kg)	93.7 (21.5)	96.2 (19.9)
BMI (kg/m^2^)	32.0 (6.8)	32.2 (5.4)
Waist circumference (cm)	100.0 (11.2)	101.5 (14.1)
HbA1c (mmol/mmol)	51.9 (10.4)	52.3 (10.0)
Total cholesterol (mmol/l)	4.2 (0.9)	4.4 (1.0)
LDL cholesterol (mmol/l)	2.2 (0.8)	2.3 (0.8)
HDL cholesterol (mmol/l)	1.3 (0.4)	1.2 (0.4)
Triglycerides (mmol/l)	2.0 (1.2)	2.5 (1.1)
Total cholesterol–HDL ratio	3.6 (1.1)	3.9 (0.9)
Systolic blood pressure (mmHg)	141.6 (18.7)	145.1 (22.2)
Diastolic blood pressure (mm Hg)	82.0 (6.9)	79.5 (8.8)
MIND diet score	7.7 (1.7)	8.3 (1.8)
Cognition		
NTB Global Cognition (z score)	− 0.07 (0.9)	0.08 (0.6)
NTB Memory (z score)	− 0.16 (0.9)	0.18 (0.9)
NTB Executive Function (z score)	− 0.05 (0.6)	0.04 (0.6)
Physical function		
Time Up and Go (seconds)	7.8 (4.4)	7.3 (1.5)
6-min walk distance (m)	476 (103)	485 (79)
Health-related quality of life		
Mental health score	48.8 (12.0)	52.2 (7.4)
Physical health score	50.4 (9.5)	51.0 (8.3)

*BMI* Body mass index, *HbA1c* glycated haemoglobin, *LDL* low-density lipoprotein, *HDL* high-density lipoprotein, *MMSE* Mini Mental State Examination, *NTB* neurocognitive test battery

### Adherence with the multidomain lifestyle intervention

**Fig. 3:**
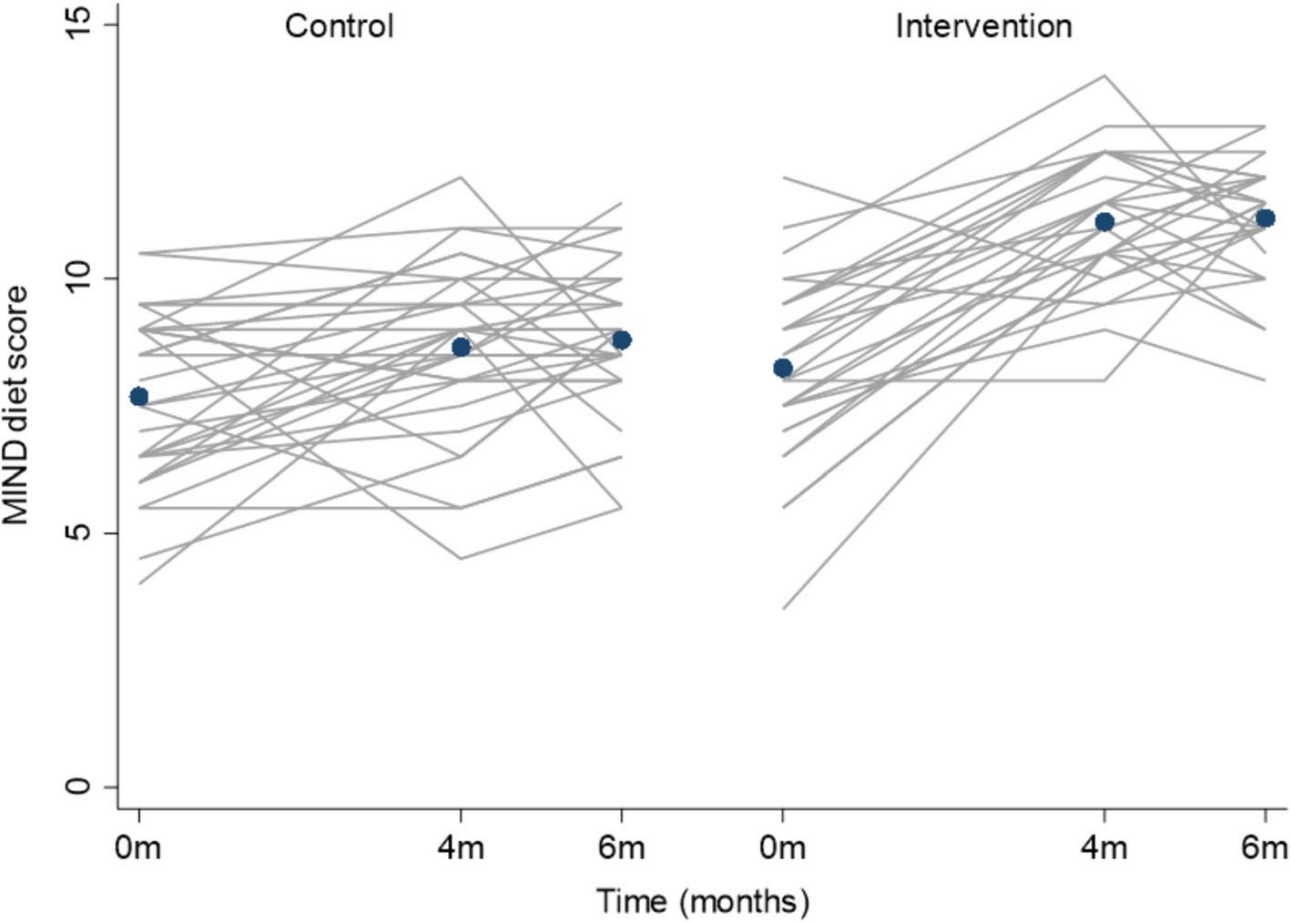
MIND diet score at each time point by group. Lines represent each individual participant MIND diet score. Dots represent the group mean MIND diet score at each time point

Participation with intervention component sessions is summarised in Table 2. Attendance was highest (> 90%) for cardiometabolic monitoring visits and with the scheduled diet sessions. All participants completed the baseline exercise prescription, and 86% completed 50% or more of their scheduled review appointments. Study records indicated that 85% of participants completed 2 or more of the home-based exercise sessions per week. Overall, adherence was lowest to the CCT schedule, with just over half the sample completing 50% or more of the expected sessions at home. There was significantly greater shift to a brain healthy diet in the intervention compared to control group (*p* < 0.001) as shown in Fig. 3 and Additional File 3. Intervention participants increased their MIND diet score by 3 points at 4 months which was maintained at 6 months (11.2 ± 1.2) and exceeded the intervention target of 2 or more points. Only 45% of participants reported their daily step count with no between-group difference in the proportion of participants reporting step count. At 6 months, daily step count increased more in the intervention group than the control group (*p* = 0.036) with intervention participants achieving ~ 9 k/day (target ~ 10 k/day) (Additional File 3).

**Table 2:** Participation with intervention component sessions

Intervention component	Completion rate (%)	Participants n (%)
Diet: 5 online sessions with nutritionist	< 50	1 (3)
	50–75	5 (17)
	> 75	23 (79)
Exercise: 17 online sessions with exercise instructor	< 50	4 (14)
	50–75	23 (79)
	> 75	2 (7)
Computerised cognitive training: 48 sessions at home	< 50	14 (48)
	50–75	3 (10)
	> 75	12 (41)
Cardiometabolic monitoring with nurse: 2 sessions	< 50	2 (7)
	50–75	–
	> 75	27 (93)

### Exploratory secondary outcomes at 6 months

The between-group differences in secondary outcomes at 6 months are shown in Table 3. The intervention and control groups had small improvements in BMI and HbA1c. There was an intervention effect for improved triglyceride level, total cholesterol–HDL ratio as well as physical and mental HR-QoL domains. Both groups showed better cognitive performance after 6 months with no indicative treatment effect on global cognition or in subdomains of memory and executive function.

**Table 3:** Between-group differences in secondary outcomes at 6 months

Outcome	Standard care control			Intervention			Diff in mean^1^ (95% CI)	*P*	Adjusted^2^ diff in mean (95% CI)	*P*
	n	Baseline	Endpoint	n	Baseline	Endpoint				
		Mean (SD)	Mean (SD)		Mean (SD)	Mean (SD)				
NTB Global Cognition (z score)	34	− 0.0 (0.6)	0.1 (0.5)	28	0.1 (0.6)	0.1 (0.5)	− 0.1 (− 0.2, 0.0)	0.10	− 0.1 (− 0.2, 0.0)	0.15
NTB Memory (z score)	34	− 0.1 (0.9)	0.6 (0.8)	28	0.2 (0.9)	0.3 (1.1)	− 0.5 (− 0.8,− 0.1)	0.01	− 0.4 (− 0.6,− 0.1)	0.02
NTB Executive Function (z score)	31	− 0.0 (0.7)	0.3 (0.5)	28	0.0 (0.6)	0.3 (0.5)	0.0 (− 0.1, 0.1)	0.92	0.0 (− 0.1, 0.2)	0.92
BMI (kg/m^2^)	36	32.0 (6.8)	31.8 (6.7)	28	32.2 (5.4)	31.6 (5.6)	− 0.3 (− 0.9, 0.4)	0.42	− 0.2 (− 0.9, 0.4)	0.471
HbA1c (mmol/mmol)	36	51.9 (10.4)	51.6 (10.6)	28	52.3 (10.0)	51.3 (10.0)	− 0.6 (− 4.5, 3.4)	0.78	− 0.7 (− 4.8, 3.4)	0.725
Triglycerides (mmol/l)	36	2.0 (1.2)	2.3 (1.5)	28	2.5 (1.1)	2.1 (1.1)	− 0.6 (− 1.1,− 0.1)	0.03	− 0.6 (− 1.1,− 0.1)	0.02
Total cholesterol–triglyceride ratio	36	3.6 (1.1)	3.8 (1.3)	28	3.9 (0.9)	3.6 (0.9)	− 0.4 (− 0.7,− 0.1)	0.01	− 0.4 (− 0.8,− 0.1)	0.01
SF-36 physical health	35	50.0 (9.5)	48.2 (9.2)	27	51.4 (8.2)	52.1 (9.1)	2.8 (− 0.1, 5.7)	0.06	3.1 (0.1, 6.2)	0.04
SF-36 mental health	35	48.3 (11.8)	47.0 (10.5)	28	52.2 (7.4)	53.8 (8.2)	3.8 (0.9, 6.7)	0.01	3.7 (0.6, 6.8)	0.02

^1^Using ANCOVA

^2^Same as ^1^ but additionally adjusting for sex, age and study site

*NTB* Neurocognitive test battery, *BMI* body mass index, *HbA1c* glycated haemoglobin, *SF-36* Short Form 36 health-related quality of life

## Discussion

The purpose of the BRAIN-Diabetes trial was to evaluate whether an adapted FINGER multidomain intervention was feasible for older adults with T2D living in rural border areas of Ireland. This population is considered at higher risk of dementia given that T2D is an established risk factor (Livingston et al. 2020) and the prevalence of dementia in rural areas is twice that of urban regions (Wiese et al. 2023). Despite significant setbacks caused by the global COVID-19 pandemic, the main findings were that the trial was feasible to deliver and efficacious in supporting individual lifestyle behavioural changes. Feasibility was demonstrated by higher-than-anticipated monthly recruitment rates within the available timescale, an overall retention rate 81% versus prespecified 75% and a between-group attrition rate 18% versus prespecified 20% threshold with more dropouts observed in the intervention than control group. The overall participation rate in the study was 67% (*n* = 79) with one-third of eligible individuals declining to participate possibly as an indirect consequence of the COVID-19 pandemic. People with T2D were disproportionally at risk of serious complications from COVID-19 infection and advised to stay at home, which led to marked decreased attendance for routine clinical care as well as participation in research activities (Khunti et al. 2022; Carr et al. 2022). Even so, we showed that recruitment via outpatient clinic services were successful for older adults with T2D in Ireland during this time and could be prioritised for future research studies.

The overall dropout rate at 6 months was 19%, which was higher than 12% reported in FINGER population (Ngandu et al. 2015) but compares well with ~ 20% dropout reported in other multidomain lifestyle intervention trials conducted in German, French and Australian populations at dementia risk (Zülke et al. 2024; Coley et al. 2019; McMaster et al. 2023). Our adherence rate was high with most participants attending > 50% of the scheduled sessions for each intervention component. The pattern of adherence rates observed across intervention components was similar to longer-term adherence rates in the FINGER trial. Comparing BRAIN-Diabetes to FINGER, the adherence rates were highest for cardiometabolic monitoring (93% vs. 95%) and diet (96% vs. 90%), followed by exercise (86% vs. 60%), and lowest for CCT (51% versus 47%). Given the short-term nature of BRAIN-Diabetes, it is possible that adherence would decrease with longer-term follow-up. Several RCTs have reported low CCT compliance among older adults (Ngandu et al. 2015; Levak et al. 2024) mainly due to limited computer literacy (Jager Loots et al. 2024) which was highlighted as a potential barrier to CCT in our population (McEvoy et al. 2023). While we attempted to overcome this barrier by enrolling those with access to a digital device for BRAIN-Diabetes, we were unable to provide the necessary one–one coaching and supervision shown to be critical for building computer self-efficacy in older adults (Richard et al. 2019). An important consideration for future study will be how to optimise cognitive stimulation for those living in rural areas who, in addition to low computer use, may also experience digital exclusion due to variable internet coverage (McEvoy et al. 2023). Emerging data indicate that paper and pen cognitive stimulation activities are both acceptable for older adults (Chew et al. 2021) and effective in enhancing cognitive performance (Georgopoulou et al. 2023) and could be a particularly useful approach for populations with low computer literacy.

Lifestyle behaviour change remains the cornerstone for self-management of T2D to prevent complications (Espeland et al. 2017) but can be challenging to achieve in practice. Furthermore, lockdown measures introduced during the COVID-19 pandemic were reportedly responsible for reduced physical activity (Elsworthy et al. 2024) and poor diet among vulnerable older UK adults (Laskou et al. 2023). Personalised support is a major determinant of compliance in diet and exercise interventions (Lemstra et al. 2018), and our results show that the provision of tailored education and incorporation of behavioural strategies such as goal setting, action planning and demonstration of exercise behaviours was efficacious in improving diet quality and increasing daily step count during the 6-month follow-up. In agreement with previous RCTs, participants also reported good adherence to the home exercise prescription (Chew et al. 2021; Ng et al. 2018; Moon et al. 2021). Target increases for MIND diet score were achieved by intervention participants and likely to have clinical benefits. For example, a 3-point increase in MIND diet score combined with modest weight loss over 3 years resulted in enhanced global cognitive function in a recent US study (Barnes et al. 2023). Furthermore, daily step count increased to ~ 9 k/day in intervention participants which has been associated with reduced dementia risk (Pozo et al. 2022) in cognitively unimpaired adults. Our exploratory analyses indicated improvement in blood lipids and HR-QoL in response to the multidomain intervention, but no treatment effect on cognitive performance. Like other multidomain trials, a placebo effect was observed on cognitive performance in control group participants which may be due to practice effects, particularly for the memory tests. Nevertheless, these results should be interpreted cautiously as BRAIN-Diabetes was not powered to detect effects on secondary endpoints and the short trial duration of 6 months is unlikely to be long enough to detect changes in cognition (Rosenberg et al. 2020).

Strengths of BRAIN-Diabetes trial include the robust a priori defined feasibility outcomes and the validated measures to assess cognitive and metabolic outcomes. A further strength is the agility of the local team to adapt to challenges posed by the COVID-19 pandemic and redesign the intervention for safe implementation in the field. The decision to deliver some intervention components remotely alongside face-to-face cardiometabolic monitoring was shown to be feasible for lifestyle behaviour change in T2D participants. This blended approach could also prove to be more economical to scale up for larger community samples due to reduced need for manpower and venue resources.

The main limitations, as mentioned above, were the short trial duration, small sample size and low statistical power to evaluate longer-term intervention effects on adherence and cognitive endpoints, which need to be the focus of a full trial evaluation. There is also the risk of selection bias in our sample who had good baseline glycaemic control and may have been more health conscious and motivated to make lifestyle behaviour changes. While the intervention was successful in promoting improved diet and walking activity, future work will consider how best to recruit and retain T2D individuals with poorer lifestyle behaviours and greater risk of accelerated cognitive decline who are likely to benefit most from changing their behaviour. Furthermore, it should be noted that behavioural diet and step count measures were based on self-reported data and subject to recall bias. It is also acknowledged that over half the sample did not return their exercise logs, and therefore, the 6-month step count results are likely based on reported data from the more motivated participants. Future work will also need to consider how best to promote non-computerised cognitive stimulation activities that are accessible and acceptable to rural populations.

In conclusion, the BRAIN-Diabetes trial showed that an adapted FINGER model was feasible for older people with T2D living in rural border areas of Ireland. The feasibility criteria for recruitment, retention and adherence to intervention components were met, and the intervention was effective at supporting the adoption of brain health lifestyle behaviours. The acceptability of the intervention was also explored with participants and will be presented separately. Overall, the learning from BRAIN-Diabetes and our experiences of conducting the pilot trial during a global pandemic will be invaluable for designing future iterations of the intervention for at-risk T2D populations.

## Supplementary Information

Below is the link to the electronic supplementary material.

Supplementary file1 (DOCX 22 KB)

Supplementary file2 (DOCX 16 KB)

Supplementary file3 (DOCX 17 KB)

## Data Availability

No datasets were generated or analysed during the current study.
