# Supplementary material for: BRAIN-Diabetes: a randomised trial to test the feasibility of an adapted FINGER multidomain intervention in adults with type 2 diabetes living in rural border regions of Ireland
Source: Eur J Ageing. 2025 Jun 24;22(1):30. doi: 10.1007/s10433-025-00862-0 (PMC12185793; doi:10.1007/s10433-025-00862-0)
Supplement: Supplementary file 1 — Supplementary file1 (DOCX 22 KB) [file 10433_2025_862_MOESM1_ESM.docx]

**Additional File 1: CONSORT Extension summary**

| **CONSERVE-CONSORT Extension: [20/02/2024]** | | | | |
| --- | --- | --- | --- | --- |
| **Item** | **Item Title** | **Description** | | |
| I. | Extenuating Circumstances | The COVID 19 pandemic caused a public health emergency across the Ireland of Ireland with lockdown measures enforced from March 2020 which were eventually eased February 2022. Recruitment for BRAIN-Diabetes was due to start in May 2021 but the start date was delayed due to COVID-19 impact on staffing levels and the changes required to the study protocol to ensure safe intervention delivery. Contracted research staff were redeployed to deliver clinical services due to increased COVID-19 infections and related illnesses which delayed capability to start data collection.  Revised protocol changes also required research ethics and governance approvals at the study sites which contributed to the delayed trial start date. | | |
| II. | Important Modifications | 1. Trial duration was reduced from 12- to 6-months to stay within budget. 2. The study protocol was revised to enable safe intervention delivery during the pandemic within available financial resources. | | |
|  |  | 1. Interventions and accompanying resources to support behaviour change were redesigned for remote delivery at the individual level. All planned group-based and face-face sessions were removed from the intervention delivery protocol. 2. Personal Protective Equipment (PPE) and risk assessment protocols were introduced for study visit assessments. 3. Home study assessments were offered to participants unwilling to travel into the dedicated research facilities. | | |
|  |  | 1. Modifications were made between June 2020-Febuary 2021 | | |
| III. | Responsible Parties | Modification decisions and budgetary changes were planned by the Chief Investigator in consultation with the scientific team. Modifications were approved by Sligo University Hospital Research Ethics Committee (775) on 9^th^ February 2021 and the Office for Research Ethics Northern Ireland (20/NI/0051) 21^st^ October 2021. | | |
| IV. | Interim data | Modifications were not informed by trial data. Recruitment and data collection had not commenced prior to protocol modifications. | | |
| **CONSORT Number and Item** | | For each row, if important modifications occurred check “direct impact” and/or “mitigating strategy” and describe the changes in the trial manuscript or supplement. Check “no change” for items that are unaffected in the extenuating circumstance. | | |
|  |  | **No Change** | **Impact*** | **Mitigating Strategy**** |
| 1 | Title and abstract | X |  |  |
| 2 | Introduction | X |  |  |
| 3 | Methods: Trial Design |  | x | Trial duration reduced from 12-months to 6-months |
| 4 | Methods: Participants |  | x | Inclusion criteria revised:   - age eligibility changes from 60+ to 50+ years - duration of T2D diagnosis expanded from within 18 months to within 5 years |
| 5 | Methods: Interventions |  | x | Intervention changes to remote delivery at the individual level. Changes to interventions:   - Exercise: a home exercise programme (ExWell@home) with online support via videos and weekly remote support from instructor for four months - Diet: Nutritional counselling delivered remotely on an individual basis at baseline and then monthly for four months - Cognitive training: instruction and demonstration offered at the study visit and then remotely x 2 sessions in the first month with a trained instructor |
| 6 | Methods: Outcomes | X |  |  |
| 7 | Methods: Sample Size | x |  | Unchanged due to the pilot design of the trial. Monthly recruitment rates monitored. |
| 8-10 | Methods: Randomisation | x |  |  |
| 11 | Methods: Blinding | x |  |  |
| 12 | Methods: Statistical methods | x |  |  |
| 13 | Results: Participant flow | x |  |  |
| 14 | Results: Recruitment |  | x | The available recruitment time was substantially reduced from 14 months to 7 months at one study site in South of Ireland and 4 months at the second study site in Northern Ireland. |
| 15 | Results: Baseline data | x |  |  |
| 16 | Results: Numbers analysed |  | x | Target sample n=140 not achieved due to shortened recruitment window |
| 17 | Results: Outcomes and estimation | x |  | The primary outcomes were feasibility of recruitment and retention, behaviour change and acceptability of the multidomain intervention and these remained unchanged for the pilot RCT. |
| 18 | Results: Ancillary analyses | N/A |  |  |
| 19 | Results: Harms | x |  |  |
| 20 | Discussion: Limitations | x |  |  |
| 21 | Discussion: Generalisability | x |  |  |
| 23 | Other information: Registration |  | x | Amendments included in the Trial Registration (ClinicalTrials.gov NCT05304975) |
| 24 | Other information: Protocol |  | x | As above |
| 25 | Other information: Funding |  | x | No additional funding available to mitigate COVID-19 impacts on study timeline and resources. |
