# Supplementary material for: BRAIN-Diabetes: a randomised trial to test the feasibility of an adapted FINGER multidomain intervention in adults with type 2 diabetes living in rural border regions of Ireland
Source: Eur J Ageing. 2025 Jun 24;22(1):30. doi: 10.1007/s10433-025-00862-0 (PMC12185793; doi:10.1007/s10433-025-00862-0)
Supplement: Supplementary file 2 — Supplementary file2 (DOCX 16 KB) [file 10433_2025_862_MOESM2_ESM.docx]

**Additional File 2: MIND Diet Component Scores**

| MIND components | Score 0 | Score 0.5 | Score 1.0 |
| --- | --- | --- | --- |
| Whole grains (serving) | <1/d | 1-2/d | ≥ 3/d |
| Green Leafy vegetables (serving) | <2/wk. | >2-<6/wk. | ≥ 6/wk. |
| Other Vegetables (serving) | <5/wk. | 5-<7/wk. | ≥ 1/d |
| Berries (serving) | <1/wk. | 1/wk. | ≥ 2/wk. |
| Red Meat and products (serving) | 7+/wk. | 6-4/wk. | < 4/wk. |
| Fish (serving) | rarely <1/mon | 1-3/mon | ≥ 1/wk. |
| Poultry (serving) | <1/wk. | 1/wk. | ≥2/wk. |
| Beans (serving) | <1/wk. | 1-3/wk. | > 3/wk. |
| Nuts (serving) | <1/mon | 1/mon-<5/wk. | ≥ 5/wk. |
| Fried/fast food (frequency) | ≥4 /wk. | 1-3 /wk. | < 1 /wk. |
| Olive oil (primary use) | Not primary oil | - | Primary oil |
| Butter/margarine (serving) | >2 serving/d | 1-2/d | <1/d |
| Cheese (serving) | ≥7/wk. | 1-6/wk. | <1/wk. |
| Pastries, Sweets (serving) | ≥7 serving/wk. | 5-6/wk. | <5 wk. |
| Wine (glass) | >1 /d or never | 1mon-6/wk. | 1 glass/d |
