# Supplementary material for: BRAIN-Diabetes: a randomised trial to test the feasibility of an adapted FINGER multidomain intervention in adults with type 2 diabetes living in rural border regions of Ireland
Source: Eur J Ageing. 2025 Jun 24;22(1):30. doi: 10.1007/s10433-025-00862-0 (PMC12185793; doi:10.1007/s10433-025-00862-0)
Supplement: Supplementary file 3 — Supplementary file3 (DOCX 17 KB) [file 10433_2025_862_MOESM3_ESM.docx]

**Additional File 3: Between group differences in MIND diet score and daily step count at 6-months.**

| Outcome | Standard Care Control | | | Intervention | | | Unadjusted^1^ | | Adjusted | |
| --- | --- | --- | --- | --- | --- | --- | --- | --- | --- | --- |
|  | n | Baseline  mean (SD) | 6-mon  mean (SD) | n | Baseline  mean (SD) | 6-mon  mean (SD) | Diff in mean  (95% CI) | P | Diff in mean  (95% CI) | P |
| MIND diet score (0-14) | 35 | 7.8 (1.7) | 8.7 (1.5) | 28 | 8.3 (1.8) | 11.2 (1.2) | 2.3 (1.6,3.0) | <0.001 | 2.2 (1.5,2.8) | <0.001 |
| Daily Step Count | 19 | 6943 (6458) | 7103  (4817) | 17 | 7209  (2727) | 9004  (3435) | 1722  (0.8, 3443) | 0.05 | 1813  (128, 3498) | 0.036 |

^1^ Using ANCOVA; ^2^ Same as ^1^ but additionally adjusting for sex, age and study site.
